# Positive effect of self-exercise following Botulinum toxin injection on the permanence of the recovery among the patients with HFS and BFS: A clinical trial

**DOI:** 10.1097/MD.0000000000038215

**Published:** 2024-06-14

**Authors:** Ümmü Serpil Sari, Mehmet Eroglu, Gülseren Büyükşerbetçi, Figen Tokucoglu, Nilay Sahin

**Affiliations:** a Balikesir University Faculty of Medicine Neurology Department, Balikesir, Turkey; b Mersin Medical Park Hospital Physical Therapy and Rehabilitation Clinic, Mersin, Turkey; c Balikesir University Faculty of Medicine Physical Therapy and Rehabilitation Department, Balikesir, Turkey.

**Keywords:** blepharospasm, botulinum toxin, hemifacial spasm, rehabilitation

## Abstract

**Background:**

Botulinum toxin (BoNT) injection serves as the primary modality for addressing hemifacial spasm (HFS) and blepharospasm (BFS), which are prevalent movement disorders affecting the craniofacial region. However, even though the short-term effectiveness of the botulinum injection may reach over 80%, the long-term effectiveness is still a debatable point Herein, we aim to investigate whether facial self-exercise following the BoNT injection can extend the time period of effectiveness.

**Methods:**

In this study, 51 volunteers who received Onabotilinumtoxin A (BoNTA) treatment for the diagnosis of HFS or BFS, were randomized into 2 groups. A detailed instruction about the self-exercise was given by an experienced physician to the subjects in Group 1. Volunteers were asked to repeat the exercise program afterward and continue to each movement for 5 seconds, to repeat each movement 10 times with a 10-second break, every day, 3 times a week for 1 week. hemifacial spasm grating scale (HSGS) and Jankovic scales were used to assess the efficacy of the treatment.

**Results:**

Both groups are similar to each other based on demographic features and the severity of the diseases. According to HSGS and Jankovic scales, at the end of the first month, there was no significant difference between the groups. At the end of the third month, the improvement achieved in the first month remained the same in each parameter in Group 1. On the other hand, in Group 2, most of the values returned to the baseline.

**Conclusion:**

Facial self-exercise following the botulinum toxin application may extend the period of effectiveness of botulinum toxin treatment the subjects with HFS and BFS.

## 1. Introduction

Hemifacial spasm (HFS) and blepharospasm (BFS) are the common clinical manifestations of the craniofacial region caused by spasms in the orbicularis oculi muscle innervated by the facial nerve. Although their pathogenesis is different, both are generally seen in women after the 4th and 5th decades.^[1]^ The clinical presentation of HFS usually starts unilaterally from the orbicularis oculi muscle and spreads to the forehead, lower half of the face and the platysma over the years. Involuntary contractions can be observed in the orbicularis oculi, frontalis, zygomaticus major, zygomaticus minor, levator labii superioris, risorius, orbicularis oris, mentalis, and platysma muscles unilaterally or bilaterally.^[2]^ BFS, on the other hand, starts with an increase in involuntary blinking and may result in the eyelid closure with involuntary contraction of the orbicularis oculi muscle. This contraction can even progress to functional blindness.^[3]^ In patients with BFS, dystonia tends to spread to adjacent parts of the body (most commonly to the oromandibular and cervical regions) compared to patients with other focal dystonias.^[4]^ In most of the cases, it is thought that the facial nerve is compressed by a vascular structure in the root exit zone region.^[5]^ Although BFS was initially considered a basal ganglia disorder, accumulating neuroimaging evidence points to anatomical and functional involvement of various brain regions, including thalamus, lower brainstem, cerebellum, midbrain, and cortex.^[6]^

It is known that systemic drug therapy has no significant efficacy in both focal movement disorders. Facial nerve root decompression with microsurgery is very effective in the treatment of HFS, however, it is a surgical intervention to posterior fossa and there is a risk of recurrence.^[7]^ On the other hand, 76% to 99% success rate was achieved with the treatment of botulinum toxin injection among HFS patients.^[8]^ The success achieved with medical treatment in BFS patients is not only short-term but also seen only in 19% to 25% of the patients.^[9,10]^

Facial nerve surgery and orbicularis oculi myemectomy are the surgical methods which are not preferred today due to their low efficacy and applicability, as well as causing serious aesthetic and functional problems.^[1]^ There is new evidence of the efficacy of deep brain stimulation applied to the internal portion of the globus pallidus and that deep brain stimulation may be helpful in selected patients with botilinumtoxin (BoNT)-resistant cranial dystonias.^[11]^

The scales are used to evaluate dystonias. It is important to rate improvement and worsening, to evaluate the patient health status and the outcome after treatment. Jankovic rating scale is a frequently used scale in HFS and BFS follow-up and studies.^[12]^ The hemifacial spasm grading scale (HSGS) is a useful tool that evaluates the localization, intensity, and frequency of HFS.^[13]^

Although the uptake of botulinum toxin takes place in the early period we hypothesized exercise which performed after the botulinum toxin injection might improve the outcome. So the frequency of toxin injection may be reduced. Consequently, the health care cost may be reduced and also the quality of life of patients may improve. To evaluate this hypothesis we aimed to investigate the effects of facial self-conducted exercises, which were initiated immediately after the botulinum toxin injection into the facial muscles and then continued regularly.

## 2. Materials and Method

This prospective study employed a randomized design according to random numbers table and included a total of 51 volunteers who received treatment with onabotulinumtoxin. Volunteers in this study were diagnosed with either HFS or BFS and received treatment at the outpatient botulinum toxin clinic of the Department of Neurology at Balikesir University Faculty of Medicine. These individuals had a history of HFS or BFS for an average duration of 1.5 to 10 years and did not have any additional neurological or eye-related conditions in their medical records. The treatment involved administering an average of 15 to 30 units of BoNTA to individuals with HFS, primarily targeting the orbicularis oculi muscle. Subjects with BFS, on the other hand, received 30 to 40 units of BoNTA. The dosage and selection of specific muscles for injection were determined by a single neurologist (USS).

Following the BoNTA injection, 24 volunteers were randomly assigned to the control (Group 2) and 27 to the study group (Group 1). Thirty minutes after BoNT injection, a single physician from the Physical Medicine and Rehabilitation Department at Balikesir University Faculty of Medicine provided instruction to the volunteers on a facial exercise program. This program focused on stretching movements specifically designed for focal dystonias (Table 1).^[14]^

**Table 1:** Facial exercises.

1. Lift your eyelids as much as you can to create wrinkles on your forehead
2. Frown
3. Close your eyes tight
4. Try to open your eyes as your eyelids closed
5. Squeeze your nose wings 6. Bring your upper lip forward
7. Pretend to whistle
8. Puff up by pulling your lower lip forward
9. Smile
10. Stretch your lips diagonally to your teeth. Pull the corners of the lips to the sides.

Since we aimed to test if the exercise may improve the outcome of BoNT injection, the volunteers were instructed to perform the exercise program regularly, repeating each movement for a duration of 5 seconds. The exercises consisted of movement of muscles which are innervated by facial muscles symmetrically (Table 1). They were advised to repeat each movement 10 times, with a 10-second break in between repetitions. This exercise routine was to be conducted daily, 3 times a week, for a total duration of 1 week.

In addition, the volunteers were given a visual and written leaflet describing how to do the exercises and an exercise diary where they would record their exercises. The control group, on the other hand, was told to continue their daily routine after BoNT injection, without any additional suggestions.

The short-term exercise diaries of the volunteers were checked in the first month of follow-up evaluation. All the volunteers who were given exercise were checked by the diaries if they exercised regularly. Both HSGS and Jankovic scales were used to assess the efficacy of the treatment which evaluates the frequency and severity of involuntary movements of mimic muscles. Patients were examined before the administration of BoNTA, in the first and third months of follow-up.

Informed consent was obtained from the participants at the beginning of the study. The ethical approval of the study was given by Ethical Committee of Balikesir University Faculty of Medicine (2021-59 on February 02, 2021).

### 2.1. Statistics

Data were analyzed using a mixed design with repeated measures ANOVA to assess the impact of intervention on 3 outcome measures. Analyses were performed via SPSS version 28 using an alpha level of 0.05 (SPSS 28.0, IBM). All test assumptions were examined and met prior to data analysis.

## 3. Results

The study included a total of 51 subjects diagnosed with HFS (N: 43) and BFS (N: 8), who underwent treatment with BoNT. The demographic characteristics of the subjects in both groups were comparable, as indicated in Table 2. Throughout the duration of the study, no side effects associated with BoNT injection, such as blurred vision, tearing, ecchymosis, ptosis, diplopia, facial weakness, or drooping of the corner of the mouth, were observed.

**Table 2:** Baseline demographic characteristics of the subjects.

HSGS	Group 1 (N: 23)	Group 2 (N: 20)	*P* value
Sex, male, N (%)	7 (30)	12 (60)	.052
Age, mean, SD, (range)	57.65 ± 11.77 (32–80)	58.25 ± 11.60 (34–78)	.87

JAN-S and JAN-F	Group 1 (N: 27)	Group 2 (N: 24)	*P* value
Sex, male, N (%)	8 (30)	13 (54)	.076
Age, mean, SD (range)	58.81 ± 11.48 (32–80)	59.79 ± 11.66 (34–81)	.88

HSGS = hemifacial spasm grading scale, JAN-F = Jancovic scale-frequency, JAN-S = Jancovic scale-severity.

Due to missing observations in HSGS scores, the effective sample size for the analysis of HSGS was 43 and the sample size for the analysis of JANM and JAN-F scales was 51.

There was no statistically significant difference between the experimental and control groups in baseline measures of HSGS (*F* = 0.031 *P* = .86), JANM (*F* = 0.096, *P* = .76), and JAN-F (*F* = 0.014, *P* = .91).

As seen in Table 3, subjects in both groups were reported to have clinical recovery in the first month of follow-up. All of the HSGS and JAN scales’ parameters in both groups improved significantly during the first month of study.

**Table 3:** Repeated measurements test results for scores from HSGS, JAN-S, and JAN-F.

		Experimental group			Control group		
		*n*	*M*	SD	*n*	*M*	SD
HSGS	Preintervention	23	7.78	1.65	20	7.70	1.42
	Postintervention	23	3.35	2.60	20	2.50	2.56
	Follow-up	23	6.13	1.87	20	7.10	1.62
JAN-S	Preintervention	27	2.78	0.51	24	2.83	0.76
	Postintervention	27	0.93	0.78	24	0.79	0.72
	Follow-up	27	2.15	0.73	24	2.75	0.74
JAN-F	Preintervention	27	2.81	0.56	24	2.79	0.83
	Postintervention	27	1.04	0.81	24	0.75	0.67
	Follow-up	27	2.26	0.71	24	2.71	0.69

HSGS = hemifacial spasm grading scale, JAN-F = Jancovic scale-frequency, JAN-S = Jancovic scale-severity.

On the other hand, when it came to the third month of follow-up, the improvement that was seen in the first month remained only in the study group. In contrast, most of the values returned to the baseline in control group (Table 3).

Repeated measures mixed ANOVA analysis was conducted to assess the impact of the intervention (experimental and control) on the outcome measures at 3 different time points: preintervention, postintervention, and follow-up. This analysis allowed for the examination of within-subject changes over time while considering the grouping factor (intervention type) as well. The results demonstrated that the time effect was statistically significant (F (1.73–70.87) = 100.13, *P* = .0001, η^2^ = 0.71), but the intervention and time interaction was not statistically significant for HSGS outcome (F (1.73–70.87) = 3.28, *P* = .050, η^2^ = 0.074). Both groups’ HSGS scores declined significantly at postintervention measure (*P* < .001). The HSGS scores of the experimental group at follow-up measure remained significantly lower than the preintervention measure (*P* < .001). Yet, the HSGS scores of the control group at follow-up measure reached to the preintervention measure level (*P* = .17), the difference between experimental and control group at follow-up measure was not statistically significant (*P* = .078).

The time effect was statistically significant for Jancovic scale-severity (JAN-S) (F (2–98) = 210.88, *P* = .0001, η^2^ = 0.81) and Jancovic scale-frequency (JAN-F) (F (2–98) = 163.29, *P* = .0001, η^2^ = 0.77) outcome measures. Both groups’ JAN-S scores declined significantly at postintervention evaluation (*P* < .001). The JAN-S scores of the experimental group at follow-up evaluation remained significantly lower than the preintervention evaluation (*P* < .001). However, the JAN-S scores of the control group at follow-up measure returned to the preintervention evaluation level (*P* = .53). JAN-F scores declined significantly at postintervention evaluation for both the experimental and control group (*P* < .001). While the JAN-F scores of the experimental group at follow-up measure remained significantly lower than the preintervention measure (*P* < .001), the JAN-F scores of the control group at follow-up measure returned to the preintervention level (*P* = .61).

**Figure 1.**
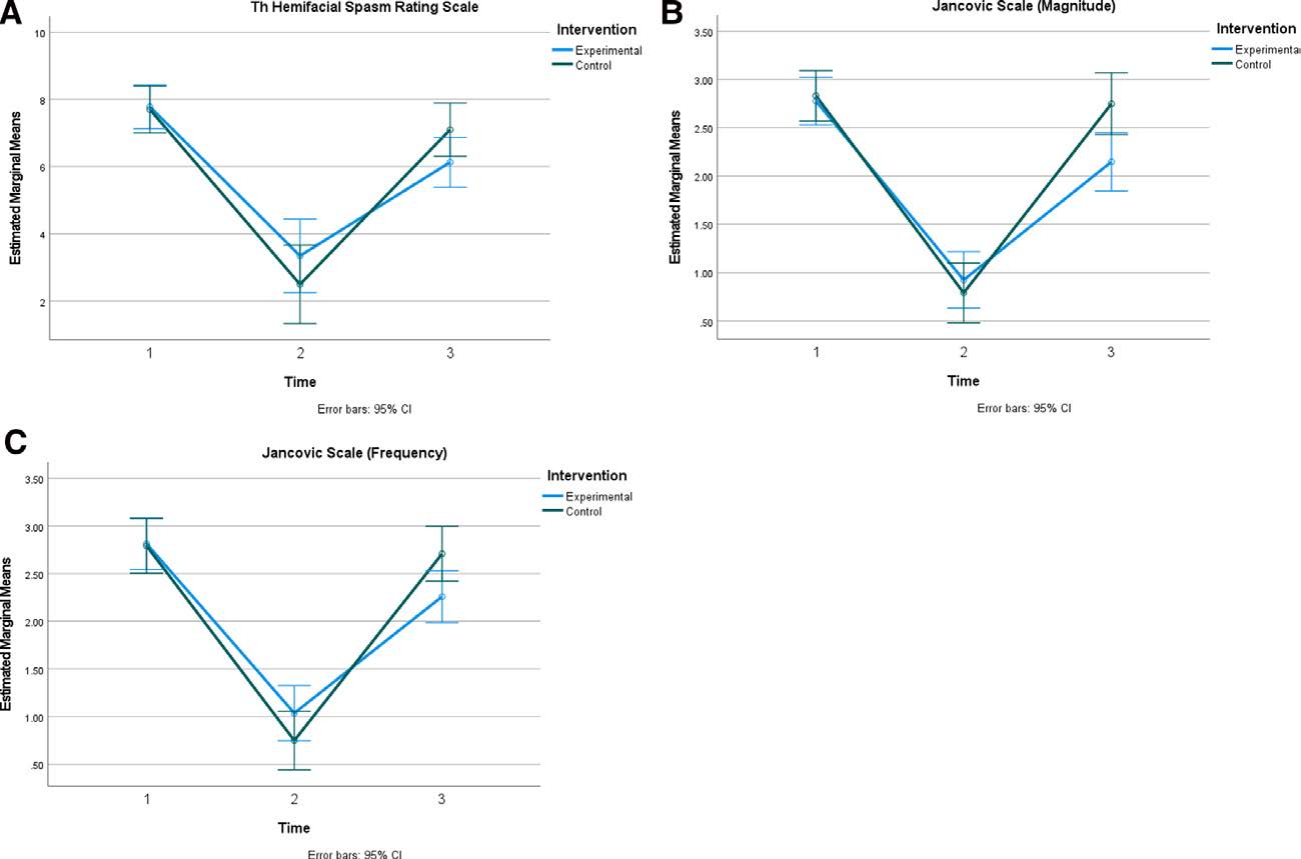
Trend in outcome measures. (A) Hemifacial rating scale. (B) Jancovic scale magnitude. (C) Jancovic scale-frequency.

The intervention and time interaction was also statistically significant for JAN-S (F (2–98) = 7.17, *P* = .001, η^2^ = 0.128) and JAN-F (F (2–98) = 5.43, *P* = .006, η^2^ = 0.10) outcome measures indicating the intervention and control groups differed significantly on post (1-month) or/and follow-up (3-month) measurements. Subsequent pairwise comparisons were conducted using least significant difference test. The results indicated that the difference between the intervention and control group was statistically significant for JAN-S (*P* = .008) and JAN-F (*P* = .027) on follow-up (3-month) measurement (see the trend in outcome measures in Fig. 1).

## 4. Discussion

In this study, the effect of a self-conducted short-term facial exercise program following BoNT application was investigated. It was observed that all 3 parameters of the Jankovic and HSGS scales showed a significant decrease compared to the baseline among the subjects who underwent short-term self-conducted exercise following botulinum toxin administration. The results indicate that there was a significant decrease in HSGS scores for both the experimental and control groups in the first month after BoNT injection. The results demonstrate a significant decrease in HSGS scores for both groups in the first month after injection. These findings provide further confirmation of the efficacy of BoNT treatment for facial movement disorders, consistent with previous reports. However, it is noteworthy that while the significant decrease in HSGS scores was sustained in the experimental group at the third month, the control group returned to their pretreatment scores.

The utilization of a superficial anesthetic preparation prior to injection, coupled with direct cold application, has been demonstrated to have an impact on the efficacy of treatment. These measures are believed to reduce the effectiveness of the treatment by decreasing the uptake of BoNT. This effect is thought to be attributed to a reduction in neurostimulation caused by the application of anesthetic and cold temperatures.^[15]^ To increase the effectiveness of BoNT, methods such as postinjection voluntary muscle contraction or electrical stimulation were evaluated. It is observed that synaptic activity may accelerate the transfer of BoNT to the presynaptic region.^[16]^ This transition was thought to affect the “binding/internalization” and “translocation” stages in particular.^[17]^ In a cross-over study involving patients with HFS, BFS, or postparalytic aberrant facial nerve regeneration, the impact of performing a 5-minute exercise immediately after BoNT application was investigated. However, the study did not reveal any significant difference in the outcomes measured between the exercise and nonexercise conditions.^[15]^ Another cross-over study that evaluated the effect of 7 minutes exercise after botulinum administration with HFS subjects found no prominent impact of the exercise either.^[18]^ On the other hand, it was reported that intense exercise in the form of out-loud reading for 1 hour after BoNT injection showed an additional affect over botulinum application with the cases with adductor spasmodic dystonia.^[19]^ Experimental physiological studies reporting that inactivity delays the toxin effect, and that electrical stimulation accelerates BoNT uptake have been available for a long time.^[20]^ Comparison of the “compound muscle action potential” amplitudes of the muscles with and without electrical stimulation after BoNT application was found to be significantly lower in those given electrical stimulation. Electrical stimulation was given during the first 24 hours after injection. There are also studies in which stimulation was performed for up to 12 weeks after BoNT application to spastic muscles. However, there was no common practice regarding stimulation intensity, frequency and location.^[17]^

In a randomized controlled study focusing on subjects with writer cramp, the effect of voluntary muscle movements after botulinum toxin administration was investigated. While 1 group was given a writing activity for 30 minutes, the other group was immobilized on the injected arm. As a result of the study, there was a significant decrease in hand and wrist muscle strength in the group that was active within the 2 to 6 weeks and third months of follow-up compared to the other group. Accordingly, it has been stated that voluntary muscle activities after BoNT injection may reduce the required dose and the incidence of side effects.^[21]^

Since it was known that the uptake of BoNT to the presynaptic region occurs in the first hours, the exercise in the previous studies was performed for a short time after the injection, unlike our study. The positive effect of postinjection exercises may not be limited to increasing uptake. Repetitive exercises may contribute to positive results, if the toxin distribution in the muscle or spread to the adjacent muscles and affect a wider area. With a long-term exercise, the feedback created by the movements may have a reducing effect on involuntary movements. Tu et al^[22]^ were able to demonstrate a gray matter volume decrease in the right inferior parietal lobule and an increase in cerebellar lobule III in the subjects with HFS compared to controls. Moreover, they showed that the volume reduction in the inferior parietal lobule increased with the longer disease duration. According to these data, the structural change in the regions associated with motor control may indicate a structural reorganization. The duration of the intervention in our study was not long enough to cause structural changes, however, it also brought to mind the idea that exercises can be effective by creating feedback. As a matter of fact, methods called neuromuscular reeducation have been used after facial paralysis.^[23]^ It was reported that the feedback provided by superficial electromyography and exercise in front of a mirror is beneficial. The aim here was to make the desired mimics and to eliminate the undesirable ones. Reeducation (neuromuscular retraining) first reduces aberrant synkinesis and then increases voluntary movement.^[24]^ After all, it is not surprising that exercise shows a similar positive effect, since there is a different level of denervation, we create with BoNT.

Today, BoNT is the first choice in treatment because its effectiveness is around 95% and the dose applied does not show a serious change over the years, and the frequency of side effects decreases as the applications continue and 10-year BoNT injection treatment in patients with HFS and BFS.^[2,10]^ Following the application of appropriate technique and adequate dose, its clinical effectiveness is observed in the first 72 hours and the clinical benefit remains for 3 to 4 months. Longer intervals and lower doses of injections will be beneficial in terms of both patient comfort and cost. Our results suggested that it would be appropriate to add facial exercise program to BoNT treatment for the continuation of the success in the treatment.

The current study has some limitations. The clinical response observed in patients with BoNT application is dependent on the experience of the practitioner. However, in our study, improvement was observed in each patient in the first month and all of the injections were done by a single neurologist. Thus, it is less likely that there is inter-investigator bias in our study. Secondly, the education and socio-cultural level of the patient may have had some effect on the answers given to the questions on the scales used.^[13]^ But, using the same scale during the study and assessing the change on the scores along the study period could downsize the effect of this issue as well. Thirdly, it can be investigated whether a regular exercise program to be carried out under the supervision of a specialist instead of relying on self-exercise would have had additional benefits. Or, the effectiveness of the exercises could have also been evaluated by providing feedback in front of a mirror or by superficial electromyography in addition to following up the scoring systems. As a future plan, it would be reasonable to observe whether the clinical benefit obtained with the applied facial exercise therapy continues in the future. The results of investigating the possible central effects of exercise with functional electrophysiological or functional imaging methods might be interesting too.

In conclusion, among the subjects with HFS and BFS, facial self-exercise following the BoNT injection may extend the time period of BoNT treatment effects. Larger studies are needed to ascertain our findings.

## Author contributions

**Conceptualization:** Ümmü Serpil Sari, Mehmet Eroglu, Figen Tokucoglu.

**Data curation:** Ümmü Serpil Sari, Nilay Sahin.

**Investigation:** Ümmü Serpil Sari.

**Methodology:** Ümmü Serpil Sari, Mehmet Eroglu, Nilay Sahin.

**Project administration:** Ümmü Serpil Sari.

**Software:** Ümmü Serpil Sari.

**Supervision:** Ümmü Serpil Sari, Figen Tokucoglu, Nilay Sahin.

**Validation:** Ümmü Serpil Sari, Gülseren Büyükşerbetçi.

**Visualization:** Ümmü Serpil Sari, Gülseren Büyükşerbetçi, Nilay Sahin.

**Writing – original draft:** Ümmü Serpil Sari.

**Writing – review & editing:** Gülseren Büyükşerbetçi, Figen Tokucoglu.
